# Combination of Immunotherapy and Radiation Therapy in Gastrointestinal Cancers: An Appraisal of the Current Literature and Ongoing Research

**DOI:** 10.3390/curroncol30070473

**Published:** 2023-07-05

**Authors:** Ritesh Kumar, Jongmyung Kim, Matthew P. Deek, Mariam F. Eskander, Prateek Gulhati, Haejin In, Timothy Kennedy, Mihir M. Shah, Miral S. Grandhi, Lyudmyla Berim, Kristen R. Spencer, Russell C. Langan, Howard S. Hochster, Patrick M. Boland, Salma K. Jabbour

**Affiliations:** 1 Department of Radiation Oncology, Rutgers Cancer Institute of New Jersey, Robert Wood Johnson Medical School, New Brunswick, NJ 08903, USA; 2 Department of Surgical Oncology, Rutgers Cancer Institute of New Jersey, Robert Wood Johnson Medical School, New Brunswick, NJ 08903, USA; me550@cinj.rutgers.edu (M.F.E.);; 3 Department of Medical Oncology, Rutgers Cancer Institute of New Jersey, Robert Wood Johnson Medical School, New Brunswick, NJ 08903, USA; 4 Division of Surgical Oncology, Department of Surgery, Emory University School of Medicine, Atlanta, GA 30322, USA; 5 Department of Medicine, Perlmutter Cancer Center of NYU Langone Health and NYU Grossman School of Medicine, New York, NY 10016, USA

**Keywords:** immunotherapy, radiation, PD L1, immune checkpoint inhibitors

## Abstract

Oncological outcomes are improving in gastrointestinal cancer with advancements in systemic therapies, and there is notable potential in combining immunotherapy and radiation therapy (RT) to allow for further improvements. Various preclinical and early phase II studies have shown promising synergy with immunotherapy and RT in gastrointestinal cancer. A few recent phase III studies have shown improved survival with the addition of immunotherapy to standard treatment for gastrointestinal cancer. The timing, duration, sequencing, and integration with other anti-cancer treatments are still areas of ongoing research. We have reviewed the published and ongoing studies of the combinations of immunotherapy and RT in gastrointestinal cancers.

## 1. Introduction

The incidence of gastrointestinal (GI) cancers is increasing worldwide, with diverse epidemiological factors and genetic and epigenetic abnormalities contributing to their development. These cancers are very common globally, and are often associated with high mortality rates [1]. Typically, patients are diagnosed at advanced stages, which poses a challenge for treatment. Although conventional treatments such as chemotherapy, radiation therapy (RT), and surgery are available, they often result in suboptimal outcomes due to local relapses and distant metastases [2]. Therefore, the exploration of innovative therapies, including immunotherapy, has great potential for treating these diseases. 

Immunotherapeutic agents have a targeted effect on malignant cells by interacting with immunogens (neoantigens) presented on them, either promoting or inhibiting immune responses [3]. A variety of immunotherapeutic modalities have been used experimentally to treat gastrointestinal (GI) cancers, including immune checkpoint inhibitors (ICI), adoptive cell transfer, chimeric antigen receptor (CAR)-T cell therapy, cancer vaccines, and/or their combinations. Immune checkpoint blockade is a widely used approach that targets various critical molecular targets. The predominant clinically utilized drugs target programmed death ligand 1 (PD-L1) found on cancer cells and antigen-presenting cells (APCs), programmed cell death protein (PD-1) present on the surface of lymphocytes, and cytotoxic T-lymphocyte associated protein-4 (CTLA-4) found on regulatory T cells (Tregs) or activated T cells. 

Various preclinical and phase II studies and one phase III study have shown promising results with ICI in GI cancers in the setting of radiation therapy. We reviewed the present status and future directions of the combination of ICI and RT in GI cancers. 

## 2. Mechanism of Radiation in Combination with Immune Checkpoint Inhibitors

The main premise for checkpoint inhibition involves inducing an immune response when pre-existing T-cells are blocked by PD-1 or CTLA-4 signaling. The programmed cell death 1 (PD-1) receptor and cytotoxic T lymphocyte-associated protein 4 (CTLA-4) are key guardians of immune checkpoints and are mainly expressed in T cells. Both were demonstrated to have a potent inhibitory role in regulating T cell responses [4]. Cancer cells detect that they are under attack from T cells by recognizing IFN-gamma, which leads to the expression of PD-L1 on the surface of cancer cells and in turn inactivates the antitumor T cell response by binding to PD-1 (CD279). CTLA-4 (CD152) is a negative regulator of co-stimulation of CD28 that is required for the activation of an antitumor T cell in a lymph node upon recognition of its specific tumor antigen, which is presented by an APC [5]. 

Radiation is a key modality of antitumor therapy and can modulate the tumor and host immuno-microenvironments [6]. Response to radiation includes the upregulation of MHC class I expression in tumor cells, and this enhances antigen recognition of cytotoxic CD8 T cells [7]. Dendritic cells play a key role in antigen presentation to T cells. RT can activate dendritic cells through the secretion of pro-inflammatory cytokines, including type I and II interferons, interleukin 1 and 2, and tumor necrosis factor-alpha [8]. Co-stimulatory molecules, including CD86 and CD70, on the surface of dendritic cells are upregulated by radiation [9]. These radiation-primed pro-inflammatory reactions induce cancer cell death and facilitate the presentation of neoantigens, which subsequently leads to improved priming and activation of dendritic cells and T cells as well [10].

The pro-inflammatory effect of radiation provides a rationale for combining radiation with immune checkpoint inhibitors (ICIs), which block the interaction between PD-1 and PD-L1 or CTLA-4 and B7-1 (CD80)/B7-2 (CD86), resulting in the activation of anti-tumorigenic T cells [11,12]. ICIs are antibodies that bind to cell receptors and are not cytotoxic in and of themselves. RT combined with ICIs may increase T cell-mediated cytotoxicity through enhancing antigen presentation and recognition, the release of pro-inflammatory cytokines, and the development of tumor antigens or neoantigens [13].

There are some potential barriers to the optimal therapeutic efficacy of RT and ICI that are being explored in future studies. These factors are intrinsic tissue sensitivity to RT, the complexities of interferon, TREX1 (DNA exonuclease) expression, and differences in abscopal effects [14].

## 3. Gastroesophageal Cancer

Programmed death ligand 1 (PD-L1) is expressed in up to 45% and 38% of esophageal and gastric cancers, respectively (at the 1% staining level) [15]. Mouse studies showed that the addition of anti-PD1 to radiation provided the greatest tumor control (both primary and contralateral non-irradiated tumors) compared to anti-PD1 with chemotherapy by increasing the ratio of CD8 T cells to Treg cells and decreasing T cell exhaustion in both the primary and contralateral implanted tumors in mice with esophageal cancers [16]. It has also been reported that RT combined with ICIs can greatly improve anti-tumor activities in radiotherapy-insensitive gastric tumor mouse models by priming the tumor microenvironment [17]. These promising results from preclinical studies led to the initiation of multiple clinical studies testing the combination of RT with ICIs in patients with gastroesophageal cancers (GECs). 

### 3.1. Phase I/II Studies

In a phase Ib study involving 19 patients with inoperable locally advanced ESCC unsuitable for chemoradiation (CRT), Zhang et al. showed that RT combined with camrelizumab, an anti-PD-1 antibody, was associated with a median overall survival (OS) of 16.7 months and progression-free survival (PFS) of 11.7 months. Patients received 60 Gy RT in 30 fractions over 5 weeks with camrelizumab (200 mg every 2 weeks) starting with RT and continuing for 32 weeks [18]. Peri-operative avelumab in combination with neoadjuvant CRT in 22 patients with stage II/III resectable esophageal and gastroesophageal junction cancers was shown to be well tolerated with no unexpected toxicities in a phase I/II study by Uboha et al. [19]. Zhu et al., in a phase Ib/II trial involving 31 patients with cT1-3N0-3M0 gastroesophageal junction (GEJ) adenocarcinoma, investigated Pembrolizumab-containing trimodality therapy, including neoadjuvant pembrolizumab-containing CRT followed by surgical resection and adjuvant pembrolizumab. This study showed acceptable tolerability, and 7/31 (22.6%) patients achieved pCR [20]. A single-arm phase II feasibility trial (PERFECT, *n* = 40) investigating neoadjuvant chemoradiotherapy (nCRT) combined with atezolizumab for resectable esophageal adenocarcinoma showed 83% of patients completed all five cycles of atezolizumab and proceeded to surgery. The pathologic complete response rate was 25% [21]. Wang et al. recently presented an interim analysis of an ongoing prospective phase II study of consolidative camrelizumab following concurrent CRT in unresectable locally advanced ESCC. The majority of patients (11/12) had stable disease with manageable toxicities [22].

### 3.2. Retrospective Studies

Wie and colleagues evaluated the addition of ICI to CRT in inoperable advanced esophageal cancer patients after first-line treatment failure in a small retrospective study. CRT plus PD-1 inhibitor was given in 26 patients and had superior OS as compared with CRT alone in 22 patients (HR 0.19, 95% CI 0.069–0.509, and *p* = 0.001), with similar PFS [23].

A retrospective study by Nie et al. using propensity score matching for patients with Stage II or higher esophageal cancer who received induction chemotherapy with ICI showed that sequential RT resulted in better PFS (15.7 vs. 5.7 months, *p* = 0.002) and OS (15.7 vs. 12 months, *p* = 0.036) than no RT [24]. Most patients received at least 4 cycles of chemotherapy and ICI (carmelizumab or pembrolizumab). RT with ICI was given to 55 patients and consisted of 60 Gy in 30 fractions. Peng et al. presented a retrospective study of 137 patients with unresectable locally advanced esophageal squamous cell carcinoma and found that induction ICI plus chemotherapy followed by definitive CRT yielded more favorable median OS (not reached vs. 25.2 months) and PFS (28.8 vs. 15.9 months, *p* = 0.128) compared with definitive CRT alone [25]. 

### 3.3. Phase III Study

CheckMate-577 (*n* = 794) showed adjuvant nivolumab increased the median DFS to 22.4 months as compared to 11.0 months (HR 0.69, *p* < 0.001) in esophageal/gastroesophageal cancer after neoadjuvant CRT and R0 resection with residual disease at the time of surgery [26]. The risk of distant recurrence or death was 26% lower, and distant metastasis-free survival was 10.7 months longer with adjuvant nivolumab than with placebo. 

### 3.4. Ongoing Studies

An ongoing phase II study (NCT03257163) is evaluating pre-operative pembrolizumab followed by adjuvant immunotherapy and CRT in Mismatch-Repair Deficient (dMMR), Epstein–Barr virus-positive, and PD-L1-positive gastric cancers. Another ongoing phase II/III study (EA2174) is evaluating preoperative nivolumab with CRT vs. pre-op CRT alone in locally advanced esophageal GEC adenocarcinoma, followed by post-surgery adjuvant ICI (nivolumab vs. nivolumab/ipilimumab).

**Figure 1:**
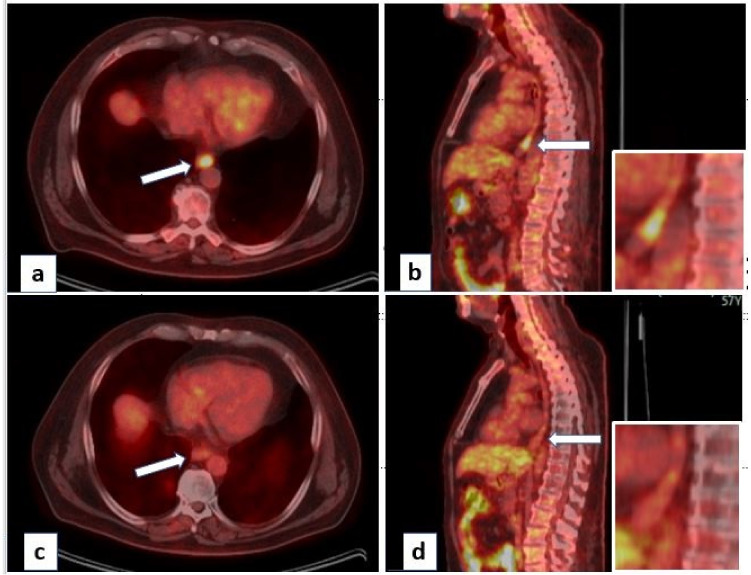
Pre-treatment (**a**,**b**) and post-treatment (**c**,**d**) PET images of a patient with cT2N1 adenocarcinoma of the lower esophagus with PDL1 30% (white arrow shows the site of esophageal disease, with the insert showing the magnified image). Patients received neoadjuvant chemoradiation (Carboplatin/Paclitaxel/RT) with concurrent nivolumab in view of high PDL1. Esophagectomy showed complete response in esophageal primary and nodes.

Published studies of the combination of ICI and RT in gastroesophageal cancers are summarized in Table 1, and selected ongoing studies are summarized in Table 2. Representative pre-treatment and post-treatment positron emission tomography (PET) images of a patient (high PDL1) who received neoadjuvant CRT (carbopaltin/paclitaxel/RT) with concurrent nivolumab are shown in Figure 1. This patient had a pCR after surgery.

## 4. Hepatocellular Carcinoma (HCC)

Hepatocellular carcinoma (HCC) is the most prevalent primary liver cancer globally [27]. Surgical resection or liver transplant in the early stages of HCC results in 5-year OS rates of 50% to 80% [28]. However, most patients are not eligible for surgical treatment due to advanced disease, poor hepatic reserve, or medical contraindications. In cases where the disease is confined to the liver, liver-directed therapies such as transarterial radioembolization (TARE) or chemoembolization (TACE), radiofrequency ablation (RFA), microwave ablation (MWA), stereotactic body RT (SBRT), or hypofractionated RT can be used as a definitive treatment, bridging therapy, or to downstage transplant eligibility [28]. Fractionated RT has yielded a response rate of 50–90% with a 1-year OS of 50–100%, which has improved with modern SBRT regimens and techniques to a 2-year local control of 70–95% [29].

Although there are various liver-directed therapies available, the survival rates for patients with unresectable HCC remain low. Furthermore, in advanced disease, multitarget tyrosine kinase inhibitors (TKI), such as sorafenib and lenvatinib, have an OS of approximately 1 year or less [30].

### 4.1. ICI alone Studies in HCC

In cases of advanced or unresectable HCC, ICI has shown improved results when compared to TKI. IMbrave150 demonstrated a 1-year OS of 67.2% with atezolizumab plus the anti-angiogenic bevacizumab compared to 54.6% with sorafenib [31]. The median PFS was 6.8 months with atezolizumab-bevacizumab and 4.3 months with sorafenib (HR 0.59, *p* < 0.001). The Himalaya 3-arm trial compared tremelimumab (anti-CTLA-4)/durvalumab (anti-PDL1) combination to durvalumab monotherapy or sorafenib alone in unresectable HCC. Most patients had Child Pugh A (approx. 98%) grade and BCLC C (approx. 80%) stages. Objective response rates were higher in the combination ICI arm as compared to the sorafenib arm (20% vs. 5%). The 3-year OS with a combination of limited-dose tremelimumab (anti–CTLA-4) and durvalumab (anti-PDL1) was significantly superior to sorafenib alone (30.7% vs. 20.2%; HR 0.78, *p* = 0.0035) [32]. Therefore, the combination of local and systemic therapies for HCC is a subject of great research interest aimed at achieving better outcomes.

### 4.2. RT with TKI in HCC

RTOG 1112 investigated the impact of adding SBRT (27.5–50 Gy in five fractions) to sorafenib in advanced HCC; however, the accrual was closed early as the standard of care for systemic therapy changed. The results of the accrued patients showed that adding SBRT improved OS (15.8 vs. 12.3 months, HR 0.77, *p* = 0.0554) and PFS (9.2 vs. 5.5 months, HR 0.55, two-sided *p* = 0.0001) in patients with advanced HCC compared to sorafenib alone. The OS was statistically significantly improved for the SBRT/sorafenib arm after adjusting for variables such as performance status, Child Pugh score, and degree of vascular invasion (HR = 0.72, 2-sided Cox *p* = 0.042). The addition of SBRT did not increase treatment-related grade 3+ adverse events [33]. 

### 4.3. RT with ICI in HCC: Phase I Study

Studies combining RT with ICIs are limited. A Phase 1 randomized trial in 14 patients with advanced or unresectable HCC compared liver SBRT (40 Gy in five fractions) followed by either nivolumab alone or nivolumab plus ipilimumab. All patients had Child Pugh A grade; four had extra-hepatic disease, and four had tumor thrombi. Two patients had received prior systemic therapy with TKI, and two had received prior liver-directed therapy. The median radiated target lesion size was 6.8 cm (range, 1.5–13.6 cm). The nivolumab plus ipilimumab arm had a better overall response (23–87% vs. 0–39%), median PFS (11.6 vs. 2.7 months), and median OS (41.6 vs. 4.7 months), which was not statistically significant. The 3-year OS with combination immunotherapy was 57%. Dose-limiting toxicities within 6 months occurred in one of six patients in the nivolumab arm and one of seven patients in the nivolumab plus ipilimumab arm. Grade 3 hepatotoxicity was seen in three patients in the combination immunotherapy arm and one patient in the nivolumab alone arm. The results showed that multimodal therapy was safe and had favorable outcomes in patients with SBRT with nivolumab plus ipilimumab [34]. 

### 4.4. RT with ICI in HCC: Retrospective Data

SBRT followed by nivolumab in unresectable HCC in a retrospective case series of five patients by Chiang et al. demonstrated two complete responses and three partial responses, and no tumor progression after a median follow-up of 14.9 months [35]. In a follow-up study of 16 patients by the same group, the use of SBRT in combination with nivolumab demonstrated a 1-year PFS of 93.3% and a 1-year OS of 93.8% [36].

### 4.5. RT with ICI in HCC: Ongoing Studies

Other ongoing trials include NCT03482102, a single-arm phase 2 trial of durvalumab and tremelimumab with SBRT; NCT03316872, a single-arm phase 2 study of pembrolizumab with SBRT; and NCT05366829, a phase 2 study of hypofractionated RT followed by tislelizumab. The published results and ongoing trials are summarized in Table 3 and Table 4, respectively. 

### 4.6. TARE with ICI in HCC

TARE with Y-90 (Yttrium-90) induces both local and systemic immune activation that corresponds to the sustained response [37]. Rivoltini et al. showed that a significant proportion of Y-90-induced CD4+ and CD8+ T cells expressed high levels of the inhibitory checkpoint marker PD-1 [38]. A phase 2 study of 42 patients with unresectable HCC treated with TARE followed by nivolumab showed 41.5% objective response rates (ORR) with a median time to progression (TTP) of 8.8 months and a median OS of 20.9 months [39]. A similar phase 2 study of 40 patients with advanced HCC treated with TARE followed by nivolumab by Tai et al. showed an ORR of 30.6% [40]. The currently undergoing ROWAN study is a prospective, multicentric, randomized, phase 2 study to assess the durability of local tumor control in HCC patients who receive TARE followed by durvalumab and tremelimumab, compared to those who receive TARE alone in HCC patients not eligible for or who have declined treatment with resection and/or ablation or liver transplant.

## 5. Cholangiocarcinoma (CCA)

Adjuvant RT (45–54 Gy in 1.8–2.0 Gy per fraction) may be used in cholangiocarcinoma (CCA) following surgery with positive margins and is considered for ≥T3 or lymph node positive disease [41,42]. The role of RT (37.5 Gy–67.5 Gy in 15 fractions) is currently being investigated in locally advanced CCA following chemotherapy as part of the NRG GI 001 trial.

### 5.1. ICI in Advanced CCA

In advanced-stage CCA, the addition of durvalumab to gemcitabine/cisplatin (GC) chemotherapy followed by maintenance durvalumab showed improved 2-year OS (24.9% vs. 10.4%; HR 0.8, *p* = 0.021) as compared to GC chemotherapy with placebo in the TOPAZ-1 (*n* = 685) clinical trial [43]. The objective response rates were increased in the durvalumab + GC arm to 26.7% as compared to 18.7% in the GC alone arm. Thus, the addition of RT to systemic therapy (including ICI) is an interesting prospect in advanced CCA and has been explored in case reports [44,45]. 

### 5.2. RT with ICI in CCA: Phase II Studies

Currently, there are a few phase II clinical trials aimed at assessing the effectiveness and safety of the combination of RT and ICI. The CORRECT trial is comparing radiation (SBRT or IMRT) plus ICI (camrelizumab) in the first line against standard chemotherapy, GC, in unresectable intrahepatic CCA (NCT03898895). Another trial is underway to investigate the combination of tislelizumab and RT (using either IMRT or SBRT) in the second-line setting post-chemotherapy with no ICI (NCT04866836). Both of these trials were designed prior to the TOPAZ-1 trial, and there is now a need for new trials to compare RT plus ICI with GC/durvalumab or post-ICI therapy. The ongoing studies are tabulated in Table 5. 

## 6. Pancreatic Cancer

Pancreatic cancer is the third most common cause of cancer mortality in the United States [27]. Advancements have been made over the years in the management of pancreatic cancer, including improvements in both systemic and local therapies, along with dose escalation with IMRT and SBRT. Neoadjuvant therapy may be employed in cases of resectable pancreatic cancer (RPC). For borderline resectable pancreatic cancer (BRPC), neoadjuvant therapy is given to enhance resection rates, achieve negative margin status, and ensure receipt of therapy, as 40% of patients do not complete adjuvant therapy after surgical resection (SWOG S1505) [46]. However, the resection rate stands at approximately 60–75% after neoadjuvant therapy, with negative margins being achieved in 60–80% of resected patients [46,47]. The addition of ICI to neoadjuvant CRT has been explored in a few small studies.

### 6.1. RT with ICI in RPC and BRPC

Rahma et al. conducted a phase II study of 37 patients with RPC and BRPC. Patients were randomized to receive either CRT alone prior to resection or concurrent pembrolizumab with CRT (50.4 Gy in 28 fractions plus Capecitabine) [48]. After neoadjuvant therapy, 9/24 (37.5%) patients in the ICI arm had unresectable disease compared to 4/13 (30.8%) patients in the control arm with similar median OS (27.8 vs. 24.3 months, *p* = 0.68). The most common grade 3+ toxicities were lymphopenia (29% vs. 31%) and diarrhea (8% vs. 0%). The initial findings indicate that the combination of CRT and Pembrolizumab was well tolerated, but its impact on the densities of TILs and other immune cell populations within the tumor was minimal. Currently, larger prospective phase II trials are being conducted to gain a better understanding of the potential benefits of combining immunotherapy with chemotherapy and RT in the neoadjuvant setting.

### 6.2. RT with ICI in LAPC

There are various challenges in delivering radiation in patients with locally advanced pancreatic cancer (LAPC) due to factors such as tumor size, location, invasion into adjacent bowel, inability to control internal motion, and limited access to on-board imaging or adaptive RT. The role of immunotherapy in combination with RT is also an active area of investigation in LAPC. Zhu et al. conducted a phase II randomized trial on 170 patients with locally recurrent pancreatic cancer after resection and adjuvant chemotherapy. There was an OS benefit in patients who received SBRT (40 Gy in five fractions), pembrolizumab, and trametinib in comparison to those who underwent SBRT (40 Gy in five fractions) in combination with gemcitabine. The median OS was 14.9 vs. 12.8 months (HR = 0.69, *p* = 0.02) [49]. 

There are other ongoing clinical trials examining the safety and efficacy of combination immunotherapy with RT in pancreatic cancer, as shown in Table 6. 

## 7. Colorectal Cancer

Colorectal cancer (CRC) is the fourth most frequently diagnosed cancer and the second leading cause of cancer death in the United States [27]. Around 5% of patients with metastatic CRC have either germline or somatic mutations in DNA mismatch repair (MMR) genes [50]. The occurrence of mismatch repair deficiency (dMMR) or microsatellite instability-high (MSI-H) is linked to DNA repair deficiency, leading to a higher mutational load. Tumors that exhibit dMMR/MSI-H have demonstrated a significant response to ICI treatment, as opposed to proficient MMR (pMMR) or microsatellite-stable (MSS) tumors due to the large number of neoantigens they express [51]. Based on the Checkmate 142 trial, the combinational therapy of nivolumab with ipilimumab is approved for metastatic CRC with dMMR/MSI-H [52]. Pembrolizumab has been demonstrated to improve outcomes in advanced metastatic CRC as a first-line setting for dMMR/MSI-H (KEYNOTE-177) [53]. The positive outcomes of ICI in mCRC have led to its exploration in non-metastatic settings as well. 

### 7.1. ICI alone in CRC: Phase II Studies

The NICHE study was a phase II study that investigated neoadjuvant ipilimumab and nivolumab for CRC with dMMR/MSI-H or pMMR/MSS. Forty patients with 21 dMMR and 20 pMMR tumors were treated with a single dose of ipilimumab and two doses of nivolumab before surgery. Out of the 20 dMMR tumors, all of them showed pathological responses, with 19 demonstrating major pathological responses (MPR) and 12 showing pathological complete responses (pCR). In the case of pMMR tumors, only 4 out of 15 exhibited pathological responses, with 3 MPR and 1 partial response [54]. The NICHE-2 study investigated the same neoadjuvant immunotherapy regimen of ipilimumab and nivolumab in 112 patients with non-metastatic dMMR advanced colon cancer. Most (89%) of patients have stage III disease, with 77% having high-risk stage III and 64% having T4 tumors. Recently presented results showed MPR in 95% of patients, including pCR in 67% of patients. At a median follow-up of 13 months (range 1–57), none of the patients had disease recurrence [55]. 

Cercek et al. conducted a phase II study on patients with dMMR/MSI-H stage II or III rectal cancer to explore the efficacy of neoadjuvant therapy with dostarlimab [56]. As part of the trial, the patients were treated with dostarlimab for a period of six months, with further plan of CRT and surgery for those with residual disease. All (*n* = 12) patients who participated in the trial achieved a complete clinical response to dostarlimab, and none of them required CRT or surgery at a minimum 6-month follow-up (range 6–25 months). 

### 7.2. RT with ICI in CRC: Phase I/II Studies

In pre-clinical studies, xenograft models of CRC have demonstrated excellent synergy between RT and ICI [57,58]. Patients with locally advanced rectal cancer were treated with CRT (50.4 Gy in 28 fractions) with capecitabine followed by nivolumab as part of the phase I/II VOLTAGE trial. Among the patients with pMMR/MSS, 30% achieved pCR, while 60% of the dMMR/MSI-H patients achieved pCR [59]. 

The NRG-GI002 trial is currently awaiting final results. It is a randomized phase II study that examines the effects of total neoadjuvant therapy (TNT) with FOLFOX treatment followed by concurrent CRT (50.4 Gy in 28 fractions) with capecitabine. In the experimental arms, pembrolizumab or veliparib was added to CRT (NCT02921256). The addition of pembrolizumab for a non-biomarker-selected group of rectal cancer patients was associated with improved 3-year OS (95% vs. 87%; HR 0.35, *p* = 0.04) and a similar 3-year DFS of 64%. Veliparib did not improve 3-year outcomes [60]. 

### 7.3. RT with ICI in CRC: Ongoing Studies

An intriguing study (NCT04304209) is currently underway to investigate the impact of sintilimab on locally advanced rectal cancer, based on MMR/MSI status. In cohort A, patients with dMMR/MSI-H will receive neoadjuvant sintilimab and undergo surgery or observation and adjuvant therapy, while in cohort B, patients with pMMR/MSS will receive neoadjuvant CRT (45–50 Gy in 25 fractions) ± sintilimab and undergo surgery or observation and adjuvant therapy.

Neo-adjuvant nivolumab/ipilimumab combination with short course pelvic RT (SCRT) 25 Gy in five fractions is currently under investigation in locally advanced rectal cancer in the phase II EOCG-ACRIN 2201 study (NCT04751370), whereas another phase II clinical trial (NCT04109755) is studying the impact of combining pembrolizumab with SCRT (25 Gy in five fractions) in the neo-adjuvant treatment of localized dMMR/MSI RC. The outcomes of these forthcoming clinical trials will provide insight into the role of radio-immunotherapy in the management of RC (Table 7). 

## 8. Anal Cancer

Anal cancer accounts for approximately 3% of all GI cancers in the United States [27]. Standard CRT (42–54 Gy in 28–30 fractions) is highly curative in treating early and locally advanced anal cancer, but about 30% of patients experience relapse or persistent disease [61]. Because of the association of HPV with anal cancer, as it is with head and neck cancer and cervical cancer, the use of ICI in locally advanced and metastatic anal cancer is being investigated [61]. 

Promising results have been obtained in patients with metastatic and surgically unresectable recurrent anal cancer who have received ICI treatment. In a multicentric phase 2 trial by Morris et al., previously treated patients with unresectable metastatic anal cancer achieved a 24% objective response rate with nivolumab, and no serious adverse events were reported [62]. Pembrolizumab was investigated in a phase 2 study, KEYNOTE-158, as a treatment for previously treated advanced anal squamous cell carcinoma. The study showed that 11% of patients had an objective response, with 15% of patients with PD-L1-positive tumors and 3% of patients with PD-L1-negative tumors responding positively. Additionally, 18% of patients experienced grade 3–4 adverse events [63].

The positive outcomes observed in patients with advanced anal squamous cell carcinoma have resulted in clinical trials that assess the potential of ICI in these patients. A completed phase III trial, ECOG-ACRIN 2165, is assessing the role of nivolumab after CRT (45–54 Gy in 30 fractions) in patients with high-risk stage II-IIIB anal cancer (NCT03233711). Patients were randomized to up to 6 months of adjuvant nivolumab vs. observation after CRT with the primary endpoint of DFS. Results are awaited for this study. The currently ongoing studies are shown in Table 8.

## 9. Limitations

The combination of RT and ICI in GI cancer is still evolving. The duration of therapy, sequencing of therapy, and treatment for recurrence after ICI are still under investigation. There should be awareness of immune-mediated complications, especially colitis, in patients receiving RT and ICI for GI cancer, and early intervention is needed to avoid high-grade toxicities. There are some potential barriers to the optimal therapeutic efficacy of RT and ICI that are being explored in future studies. The collection of quality of life (QOL) data is also ongoing. 

## 10. Conclusions

The combination of radiation therapy and immune checkpoint inhibitors is increasingly being tested to improve oncological outcomes in gastrointestinal cancers. The available published studies show encouraging results with acceptable toxicity profiles. The optimal timing of RT and ICI is still evolving. Currently, ICI after RT has shown the most benefit, as in Checkmate 577 in esophageal cancer. There is an increasing interest in RT and ICI in dMMR/MSI-H CRC and gastric cancers, as well as in the neoadjuvant or pre-operative setting. The results of the ongoing prospective studies will determine the role of the combination of ICI and RT in GI cancers. 

## Figures and Tables

**Table 1:** Gastro-esophageal cancer (published studies).

Author/Study	Type of Study	Number (*n*)	Disease Status	ICI	Intervention	Results
Zhang [18]	Phase 1b	19	Locally advanced	Camrelizumab	RT-ICI f/b ICI	PFS 11.7 months OS 16.7 months
Zhu [20]	Phase 1/2	31	Resectable (Stage II/III)	Pembrolizumab	CRT-ICI f/b Surgery f/b adjuvant ICI	pCR in 22.6%
PERFECT [21]	Phase 2	40	Resectable	Atezolizumab	CRT-ICI f/b Surgery	pCR in 25%
Wang [22]	Phase 2	12	Locally advanced	Camrelizumab	Definitive CRT f/b consolidative ICI (*n* = 12)	11/12 patients had SD
Wie [23]	Retrospective	55	Inoperable	Camrelizumab Tislelizumab Sintilimab	CRT-ICI (*n* = 26) CRT alone (*n* = 29)	Improved OS with CRT-ICI
Nie [24]	Retrospective	134	Locally advanced	Carmelizumab Pembrolizumab	CHT-ICI f/b RT (*n* = 55) CHT-ICI (*n* = 79)	PFS (15.7 vs. 5.7 m) OS (15.7 vs. 12 m)
Peng [25]	Retrospective	62	Locally advanced	----	CHT-ICI f/b definitive CRT	PFS 28.8 months
CheckMate 577 [26]	Phase 3	794	Resectable	Nivolumab	NA-CRT f/b Surgery +/−adjuvant ICI (*n* = 532 vs. 262)	DFS 24.4 vs. 11 months

**Table 2:** Gastro-esophageal cancer (ongoing studies).

NCT Number	Interventions	Primary Outcome	Phase
NCT05650216	Camrelizumab + CRT	Safety, pCR	2
NCT05043688	Camrelizumab + CRT	pCR	2
NCT04229459	Nivolumab + CRT	pCR	2
NCT03777813	Durvalumab +CRT vs. CRT	PFS	2
NCT05520619	Tislelizumab + CRT	PFS	2
NCT05387681	Envafolimab + CRT	pCR	2
NCT04929392	Pembrolizumab + CRT	pCR	2
NCT04888403	Toripalimab + CRT	pCR	2
NCT03257163	Pembrolizumab → Surgery → adj CHT and CRT with Pembrolizumab	DFS	2
NCT04973306	Tislelizumab + CRT vs. CRT	pCR, OS	2, 3
NCT03604991	Pre-op Nivolumab + CRT vs. Pre-op CRT with post-surgery adjuvant (Nivo vs. Nivo/Ipi)	pCR, DFS, OS	3
NCT04404491	Camrelizumab + RT vs. RT + CHT	AE, PFS	3
NCT04821843	Nimotuzumab + CRT vs. Nimotuzumab + CHT	OS	3
NCT04821778	Nimotuzumab + CRT vs. CRT	OS	3
NCT05244798	Sintilimab + CHT vs. Sintilimab + CRT vs. CRT	pCR	3
NCT04807673	Pembrolizumab + CRT	Event-Free Survival (EFS)	3

**Table 3:** HCC published results.

Author	Type of Study	Patient Characteristics	Intervention	Results
Chiang [35]	Case series	*N* = 5 Unresectable HCC	SBRT + Nivolumab	CR: 2/5 PR: 3/5
Chiang [36]	Retrospective	*N* = 16	SBRT + Nivolumab	CR: 50% PR: 37.5%
Juloori [34]	Prospective Phase 1 RCT	*N* = 14	SBRT + Nivolumab (*n* = 6)	PR—12.5% SD—37.5% PD—50%
			SBRT + Ipilimumab + nivolumab (*n* = 8)	PR—50% SD—37.5% PD—12.5%

**Table 4:** HCC (ongoing studies).

NCT Number	Interventions	Outcome Measures	Phase
NCT05488522	SBRT + atezolizumab and bevacizumab	PFS	1
NCT03817736	TACE followed by SBRT followed by Avelumab	Response Rate/Amenable to surgery	2
NCT04913480	SBRT + Durvalumab (1 yr)	PFS	2
NCT04988945	TACE followed by SBRT followed by Durvalumab + Tremelimumab	Response Rate/Amenable to surgery	2
NCT04611165	Hypofractionated radiation (10 fractions) + Nivolumab	PFS	2
NCT04430452	Hypofractionated radiation + Durvalumab +/− Tremelimumab	Response Rate	2
NCT03316872	SBRT + Pembrolizumab	Response Rate	2
NCT05366829	RT + Tislelizumab	PFS	2
NCT04167293	SBRT + Sintilimab	PFS	2/3

**Table 5:** CCA ongoing studies.

NCT Number	Interventions	Outcome Measures	Phase
NCT04708067	RT + Bintrafusp Alfa	Response	1
NCT04866836	RT + Tislelizumab	Response	2
NCT03898895 (CORRECT)	RT + Camrelizumab	PFS	2

**Table 6:** Ongoing studies in pancreatic cancer.

NCT Number	Disease Status	Interventions	Outcome Measures	Phase
NCT04098432	Locally Advanced Unresectable Pancreatic Adenocarcinoma	SBRT + Nivolumab	Safety	1/2
NCT04247165	Pancreatic Cancer	SBRT + Ipilimumab + Nivolumab	PFS	1/2
NCT04390399	Locally Advanced or Metastatic Pancreatic Cancer	SBRT + Chemo +/− IT	PFS/ORR	2
NCT04361162	MSS Pancreatic Cancer	RT + Nivolumab + Ipilimumab	ORR	2
NCT03563248	Localized Pancreatic Cancer	FOLFIRINOX + SBRT + Surgery +/− Nivolumab +/− Losartan	R0 Resection	2
NCT05116917	Pancreatic Cancer	SBRT + Nivolumab + Influenza Vaccine	ORR	2
NCT03161379	Borderline Resectable Pancreatic Cancer	SBRT + Nivolumab + GVAX Pancreas Vaccine	ORR	2

**Table 7:** Rectal cancer ongoing studies.

NCT Number	Phase	Stage	ARM	Interventions	Outcome Measures
NCT03127007 (R-IMMUNE)	Phase 1/2	LARC	A	LC CRT + Atezolizumab → TME	AE, pCR
			B	LC CRT → TME	
NCT02948348	Phase 1/2	LARC	--	LC CRT + Nivo → TME	pCR
NCT05245474	Phase 2	LARC	A	LC CRT + Concurrent Tislelizumab → TME	pCR
			B	LC CRT + Sequential Tislelizumab → TME	
			C	LC CRT → TME	
NCT05576480	Phase 2	LARC	--	SCRT → Penpulimab + CAPEOX → TME	pCR
NCT05086627	Phase 2	LARC	A	SCRT → Tislelizumab + CAPEOX → TME → CAPEOX	pCR
			B	SCRT → CAPEOX → TME → CAPEOX	
NCT04621370 (PRIME-RT)	Phase 2	LARC	A	SCRT + Durvalumab → FOLFOX	pCR, cCR
			B	LCRT + Durvalumab → FOLFOX	
NCT05507112	Phase 2	LARC	A	LC CRT + Concurrent Tislelizumab → TME	pCR
			B	LC CRT → TME	
NCT04503694	Phase 2	LARC	--	Regorafenib + Nivolumab → SCRT → Regorafenib + Nivolumab → TME → +/− adjuvant Chemo	pCR
NCT04751370	Phase 2	LARC	--	Nivo/Ipi → SCRT → Nivo/Ipi → TME	pCR
NCT03921684	Phase 2	LARC	--	LC CRT → FOLFOX + Nivolumab → TME	pCR
NCT04124601	Phase 2	LARC	A	LC CRT	AE, Response
			B	LC CRT → Nivo/Ipi	
NCT03299660	Phase 2	LARC	--	LC CRT → Avelumab → TME	pCR
NCT03854799	Phase 2	LARC	--	LC CRT → Avelumab → TME	pCR
NCT03503630	Phase 2	LARC	--	SCRT → Avelumab + FOLFOX → TME	pCR
NCT04293419 (DUREC)	Phase 2	LARC	--	FOLFOX + Durvalumab → LCCRT → TME	pCR
NCT05009069	Phase 2	LARC	A	LC CRT + Atezolizumab + Tiragolumab → TME	pCR
NCT05484024	Phase 2/3	LARC	A	SCRT → NACT + Sintilimab → W/W or TME	pCR, DFS
			B	SCRT → NACT → W/W or TME	

**Table 8:** Anal cancer ongoing studies.

NCT Number	Phases	Stage	Interventions	Outcome Measures
NCT04046133 (CORINTH)	Phase 1	LA III A/B	CRT + Pembrolizumab	AE, Response
NCT04230759 (RADIANCE)	Phase 2	LA IIB-IIIC	CRT (with 5FU/MMC)	DFS
			CRT (with 5FU/MMC/Durvalumab)	
NCT04929028	Phase 2	Low Risk HIV	CRT (with 5FU/MMC)	AE, DFS
		High Risk HIV	CRT (with 5FU/MMC/Nivolumab)	
NCT05661188 (TIRANUS)	Phase 2	I-IIIB	CRT (with 5FU/MMC/Tiraglolumab/Atezolizumab)	cCR
NCT03233711	Phase 3	LA II-IIIB	CRT	DFS
			CRT → Nivolumab	
NCT05374252	Phase 3	LA III	CRT (with 5FU/MMC)	PFS, OS, cCR
			CRT (with 5FU/MMC/Sintilimab) → Adjuvant Sintilimab	
